# The Diagnostic and Clinical Significance of Anti-Mutated Citrullinated Vimentin Antibodies in Rheumatoid Arthritis-Associated Interstitial Lung Disease: A Scoping Review

**DOI:** 10.3390/antib15040060

**Published:** 2026-07-13

**Authors:** Christian D’Elia, Giada Santagata, Serena Guiducci, Holger Bang, Mariangela Manfredi, Maria Infantino, Maurizio Benucci

**Affiliations:** 1 Department of Experimental and Clinical Medicine, Division of Rheumatology, University of Florence, 50134 Florence, Italy; christian.delia@unifi.it (C.D.);; 2 Sebia, Carl-Zeiss-Straße 49-51, 55129 Mainz, Germany; 3 Immunology and Allergology Laboratory Unit, S. Giovanni di Dio Hospital, Azienda USL-Toscana Centro, 50143 Florence, Italy; 4 Rheumatology Unit, S. Giovanni di Dio Hospital, Azienda USL-Toscana Centro, 50143 Florence, Italy

**Keywords:** rheumatoid arthritis, interstitial lung disease, anti-mutated citrullinated vimentin antibodies, anti-MCV

## Abstract

Rheumatoid arthritis-associated interstitial lung disease (RA-ILD) is one of the most severe extra-articular manifestations of rheumatoid arthritis (RA), requiring reliable biomarkers for early detection. This scoping review synthesized current evidence regarding the diagnostic performance and clinical associations of anti-mutated citrullinated vimentin (anti-MCV) antibodies in patients with RA-ILD. A comprehensive literature search was conducted across PubMed/MEDLINE, Embase, Scopus, and the Cochrane Library. Following systematic screening, two observational studies met the predefined inclusion criteria. Both included studies reported significantly higher anti-MCV positivity rates and/or serum levels in patients with RA-ILD compared with RA patients without pulmonary involvement. Specifically, one study identified an independent association between anti-MCV positivity and RA-ILD, while the other demonstrated significant correlations between anti-MCV titers and pulmonary function impairment, as well as disease activity markers. However, substantial heterogeneity was observed across the studies regarding assay platforms, positivity thresholds, and diagnostic cut-offs, which limits the direct comparability of results. While anti-MCV antibodies represent promising candidate biomarkers for RA-ILD, current evidence remains limited and is insufficient to establish definitive diagnostic, prognostic, or pathogenic significance. Consequently, larger, prospective, and multi-center studies utilizing standardized anti-MCV assay protocols are necessary to rigorously evaluate the clinical utility of these antibodies in the management of RA-ILD.

## 1. Introduction

Rheumatoid arthritis (RA) is a chronic autoimmune disease primarily affecting the joints, but it can manifest with various extra-articular complications, among which interstitial lung disease (ILD) represents a significant cause of morbidity and mortality [1,2].

RA-ILD constitutes one of the most severe extra-articular manifestations of RA, with prevalence estimates exhibiting considerable variability from 1% to 67% across studies, largely attributable to differences in diagnostic modalities such as high-resolution computed tomography (HRCT) versus clinical assessment [3,4,5].

**Figure 1:**
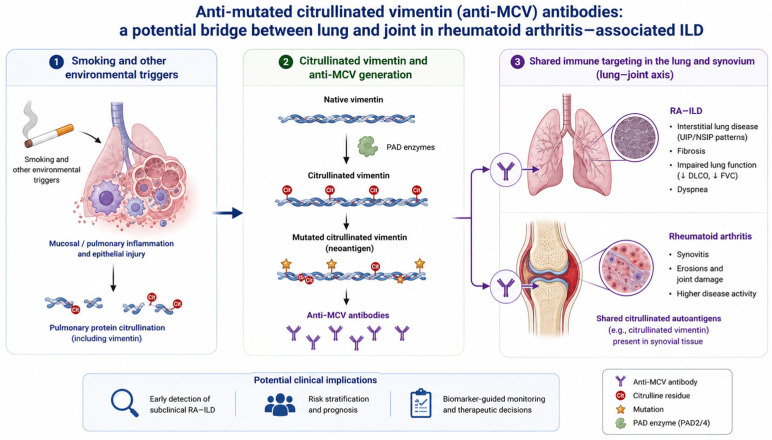
Proposed role of (anti-MCV antibodies in the lung–joint axis of Rheumatoid Arthritis-associated Interstitial Lung Disease (RA-ILD). Environmental and mucosal triggers such as smoking may promote pulmonary citrullination of vimentin, which may be associated with anti-MCV generation and shared autoimmune targeting of pulmonary and synovial tissues. These processes are hypothesized to contribute to, rather than prove causation of, both interstitial lung disease and articular manifestations in RA. The figure was created by the authors with the support of artificial intelligence-assisted graphic generation tools and subsequently reviewed and modified manually.

This condition may markedly increase morbidity through progressive respiratory impairment and diminished quality of life. In patients with clinically significant or progressive RA-ILD, mortality is substantially increased, with RA-ILD accounting for approximately 10–20% of deaths in RA cohorts and historical studies reporting a median survival of 3–7 years following diagnosis. However, prognosis is heterogeneous and depends on factors such as disease severity, radiological pattern, and the inclusion of subclinical cases detected by HRCT [6,7,8,9,10,11]. Prognostically, histological and radiological patterns significantly influence outcomes, with the usual interstitial pneumonia (UIP) subtype associated with shorter survival and worse prognosis compared with nonspecific interstitial pneumonia (NSIP) or organizing pneumonia (OP) [12,13,14,15]. Given the profound impact of RA-ILD on patient outcomes, there is a pressing need for the identification of novel biomarkers that can facilitate early diagnosis, predict disease progression, and guide therapeutic strategies. In this context, evidence suggests that anti-citrullinated protein antibodies (ACPAs) are associated with RA-ILD risk and phenotype, including greater prevalence, extent of lung involvement on HRCT, UIP pattern, and poorer prognosis [16,17,18,19,20]. Among ACPAs, anti-MCV antibodies have been proposed as candidate biomarkers for RA-ILD because higher positivity rates and titers have been reported in affected patients compared with RA patients without ILD, in some studies independently of anti-cyclic citrullinated peptide (anti-CCP) levels [21]. Vimentin is an intermediate filament protein expressed by mesenchymal cells, macrophages, fibroblast-like synoviocytes, and other cell types relevant to lung and synovial inflammation. The established MCV concept derives from anti-Sa biology and from the characterization of MCV in RA synovial fluid: Bang et al. identified human MCV isoforms, showed that vimentin antigenicity is influenced by both sequence mutation and PAD-mediated citrullination, and used recombinant human MCV as the antigen for a standardized anti-MCV ELISA [22,23,24]. Unlike anti-CCP assays, which use selected synthetic cyclic citrullinated peptides, anti-MCV assays target recombinant MCV, a full-length vimentin-derived antigen carrying defined mutated and citrullinated epitopes. In the original RA validation work, anti-MCV showed high specificity, greater sensitivity than anti-CCP in the study cohort, and correlation with Disease Activity Score in 28 joints (DAS28); however, such diagnostic or prognostic advantages vary across cohorts and assay platforms and should not be generalized to RA-ILD without disease-specific validation [22,25,26,27,28,29,30]. Several RA cohorts have linked anti-MCV positivity and elevated titers to increased disease activity (e.g., higher DAS28 scores), greater joint damage (e.g., erosions and Larsen scores), and extra-articular manifestations, although the degree of independence from other ACPAs and Rheumatoid Factor (RF) varies by study design and assay [21,31,32]. Early identification of RA-ILD remains challenging due to nonspecific symptoms such as progressive dyspnea and dry cough, which frequently overlap with other pulmonary conditions or are mistakenly attributed to aging, fatigue, or comorbid conditions, thereby contributing to diagnostic delay [33]. Risk-stratified screening has become more structured with the 2023 American College of Rheumatology (ACR)/American College of Chest Physicians (CHEST) guidance: in people with systemic autoimmune rheumatic diseases, including RA, who are at increased risk of ILD, Pulmonary Function Tests (PFTs) and HRCT of the chest are conditionally recommended for screening, whereas chest radiography, bronchoscopy, and surgical lung biopsy are not recommended as routine screening tests [34]. Conventional inflammatory markers, including C-reactive protein (CRP) and erythrocyte sedimentation rate (ESR), correlate poorly with lung involvement and cannot replace pulmonary assessment [33,35]. Consequently, reliable biomarkers for detecting subclinical disease or predicting progression in RA-ILD are currently lacking [3,21]. This gap underscores the critical need for novel serological markers to facilitate early detection and risk stratification of RA-ILD, particularly given that subclinical forms of the disease are present in approximately 30–55% of asymptomatic individuals and frequently progress radiologically over time [17,36,37,38]. This paucity of reliable biomarkers has spurred interest in pulmonary mechanisms underlying RA autoimmunity, wherein environmental exposures such as cigarette smoke may induce peptidylarginine deiminase expression and citrullination of lung proteins—such as vimentin—generating neoantigens that elicit ACPA responses that may precede articular disease and are associated with RA-ILD [39]. Emerging evidence supports a lung-centric model of disease initiation, with subclinical alveolar inflammation, epitope spreading of ACPAs, and autoantibody detection in bronchoalveolar lavage potentially contributing to both joint and interstitial pathology [40]. This notion aligns with the “lung-joint axis” hypothesis in RA pathogenesis, whereby the lung emerges as a critical mucosal site for initiating and perpetuating autoimmune responses through protein citrullination [40,41,42,43] (Figure 1).

Citrullinated vimentin identified in pulmonary tissues may thus represent a key immunological target bridging pulmonary inflammation to articular pathology [44,45]. Anti-MCV antibodies, recognizing MCVas a defined vimentin-derived ACPA specificity, therefore warrant scrutiny as candidate biomarkers for RA-ILD [21]. Although accumulating evidence implicates ACPAs in the increased prevalence, severity, and progression of RA-ILD [3,20,35], the specific diagnostic and clinical significance of anti-MCV antibodies in this context has not been comprehensively summarized, with primary studies remaining limited [21]. Therefore, the objective of this scoping review is to map and summarize the available evidence regarding the diagnostic and clinical significance of anti-MCV antibodies in RA-ILD.

## 2. Methods

### 2.1. Study Design

This study was conducted as a scoping review to map and summarize the available evidence regarding the role of anti-MCV antibodies in RA-ILD.

### 2.2. Protocol and Reporting Framework

The review methodology was informed by the methodological framework proposed by Arksey and O’Malley [46] and further refined by Levac et al. [47] and the Joanna Briggs Institute guidance for scoping reviews [48]. Reporting was conducted according to the Preferred Reporting Items for Systematic reviews and Meta-Analyses extension for Scoping Reviews (PRISMA-ScR) [49].

### 2.3. Eligibility Criteria

Studies were considered eligible if they evaluated the role, prevalence, diagnostic performance, or clinical associations of anti-MCV antibodies in patients with RA-ILD.

Original studies involving adult patients with RA and ILD were included. Studies evaluating anti-MCV antibodies, citrullinated vimentin, or closely related anti-vimentin/citrullinated vimentin antibody specificities in the context of RA-ILD were considered eligible.

We excluded reviews, editorials, conference abstracts superseded by full publications, animal or preclinical studies, studies not specifically focused on RA-ILD, and studies evaluating autoantibodies other than anti-MCV. Articles not available in English were also excluded.

No restrictions on study design were applied.

### 2.4. Information Sources and Search Strategy

A comprehensive literature search was conducted on 4 May 2026, in PubMed/MEDLINE, Embase, Scopus, and the Cochrane Library. Additional records were identified through reference mining of the included studies. No additional grey literature sources were systematically searched. The search strategy was developed and refined with the support of experienced Health Sciences librarians.

The search strategy combined terms related to RA, ILD, and anti-MCV antibodies, with mutated citrullinated vimentin used consistently as the expanded MCV term. Broader search strategies including generic ACPA-related terms were considered; however, the search was intentionally focused on anti-MCV antibody specificities to maintain conceptual consistency with the objectives of the review. Controlled vocabulary terms and free-text keywords were adapted for each database.

The PubMed search strategy was:

(“Arthritis, Rheumatoid” [Mesh] OR “rheumatoid arthritis” [tiab]) AND (“Lung Diseases, Interstitial” [Mesh] OR “interstitial lung disease*” [tiab] OR “ILD” [tiab] OR “pulmonary fibrosis” [tiab]) AND (“Vimentin” [Mesh] OR vimentin[tiab] OR “citrullinated vimentin” [tiab] OR “mutated citrullinated vimentin” [tiab] OR “mutated and citrullinated vimentin” [tiab] OR “anti-citrullinated vimentin” [tiab] OR “anti-MCV” [tiab] OR “anti MCV” [tiab] OR “anti-MCV antibodies” [tiab] OR “anti-mutated citrullinated vimentin” [tiab] OR “anti mutated citrullinated vimentin” [tiab] OR “anti-Sa” [tiab] OR “anti Sa” [tiab] OR “Sa antigen” [tiab] OR “anti-Sa antibodies” [tiab]).

Equivalent search strategies were adapted for Embase, Scopus, and the Cochrane Library.

### 2.5. Study Selection

All identified records were imported into reference management software, and duplicate records were removed prior to screening. Titles and abstracts were independently screened by two reviewers (C.D. and G.S.) according to the predefined eligibility criteria. Full-text assessment was subsequently performed independently by the same reviewers. Any disagreements regarding study eligibility were resolved through discussion with a third reviewer (M.B.).

Studies that evaluated anti-MCV antibodies in the context of RA-ILD were included.

**Figure 2:**
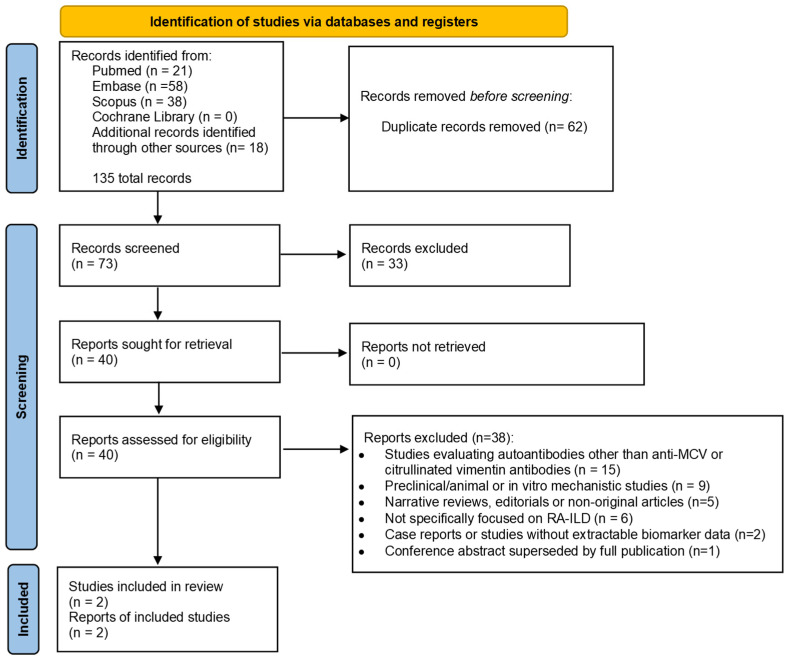
PRISMA flow diagram of study selection process. Flow diagram summarizing the identification, screening, eligibility assessment, and inclusion of studies evaluating the role of anti-MCV antibodies in RA-ILD). The literature search yielded 135 records, of which two studies met the inclusion criteria and were included in the final review.

The study selection process is summarized in the PRISMA flow diagram (Figure 2).

### 2.6. Data Charting Process

Data from the included studies were extracted using a predefined data charting form developed by the authors. Extracted information included study characteristics, study design, population characteristics, anti-MCV assessment methods, ILD-related findings, principal results, and study limitations.

The data charting process was performed iteratively and refined during the review process to ensure consistency and relevance to the review objectives.

### 2.7. Synthesis of Results

Extracted data were synthesized descriptively and summarized narratively according to the objectives of the review. Study characteristics and principal findings were organized into evidence tables focusing on the association between anti-MCV antibodies and RA-ILD.

## 3. Results

### 3.1. Included Studies

The database search yielded 117 records from PubMed/MEDLINE (*n* = 21), Embase (*n* = 58), and Scopus (*n* = 38), whereas no records were identified through the Cochrane Library. An additional 18 records were identified through reference screening, resulting in a total of 135 records. After duplicate removal, 73 records underwent title and abstract screening, and 40 full-text articles were assessed for eligibility. Following full-text evaluation, two studies met the inclusion criteria and were included in the final review.

### 3.2. Characteristics of Included Studies

Two observational studies specifically evaluating the association between anti-MCV antibodies and RA-ILD were included in the review. The included studies were published between 2016 and 2024 and adopted cross-sectional or case–control designs.

Overall, the studies evaluated serum anti-MCV antibody levels in patients with RA-ILD compared with RA patients without ILD. Both studies assessed the potential diagnostic and clinical significance of anti-MCV antibodies in relation to pulmonary involvement and disease-related characteristics.

The main characteristics and findings of the included studies are summarized in Table 1.

In a cross-sectional study involving 75 patients with RA, Tian et al. [50] evaluated the diagnostic utility of anti-MCV antibodies in identifying interstitial lung disease. The cohort comprised 37 patients with RA-ILD and 38 RA patients without pulmonary involvement. The investigators reported that both the positivity rate and serum titers of anti-MCV antibodies were significantly elevated in the RA-ILD group compared to the RA-only controls. In contrast, no significant differences were observed between the two groups regarding the prevalence or levels of anti-cyclic citrullinated peptide (anti-CCP) antibodies. Furthermore, logistic regression analysis identified anti-MCV antibodies as a potential predictor of ILD in RA patients. Given the small sample size and cross-sectional design, this should be interpreted as an association signal rather than evidence of diagnostic superiority over anti-CCP assays.

Elsayed et al. [21] conducted a case–control study including 80 patients with RA, of whom 40 had RA-ILD and 40 did not present pulmonary involvement, together with 40 healthy controls. ILD was diagnosed by HRCT, while pulmonary involvement was further assessed through pulmonary function tests, including DLCO, FVC, FEV1, and TLC. Serum anti-MCV antibody levels were significantly higher in RA patients compared with healthy controls and, importantly, in RA-ILD patients compared with RA patients without ILD. Patients with RA-ILD also exhibited significantly higher disease activity scores, inflammatory markers, Larsen scores, RF, and ACPA levels, together with significantly impaired pulmonary function parameters. Correlation analysis demonstrated that anti-MCV antibody levels were positively associated with DAS28, Larsen score, ESR, and ACPA levels, while negative correlations were observed with DLCO and FVC. Logistic regression analysis identified age, disease duration, anti-CCP levels, anti-MCV levels, and anti-MCV positivity as variables associated with ILD in that cohort. Receiver operating characteristic (ROC) curve analysis showed moderate-to-good discrimination for RA-ILD, with an AUC of 0.815, sensitivity of 80%, and specificity of 75% at a study-specific cut-off value of 155.5 U/mL. This cut-off should be interpreted as assay- and cohort-specific, particularly because positivity in the same study was defined as >20 U/mL using a Cusabio ELISA, whereas Tian et al. used an ORGENTEC anti-MCV ELISA with a ≥ 20 U/mL positivity threshold.

## 4. Discussion

The currently available evidence regarding anti-MCV antibodies in RA-ILD remains limited, with only two small, non-longitudinal observational studies specifically addressing this association (77 RA-ILD patients and 78 RA patients without ILD across both studies) [21,50]. Nevertheless, the findings are relatively consistent in indicating a link between anti-MCV positivity or elevated titers and pulmonary involvement in patients with RA. Although preliminary, these observations are biologically plausible and underscore the value of further targeted investigation into anti-MCV antibodies as potential biomarkers in RA-ILD. Rather than supplanting conventional anti-CCP assays, anti-MCV measurement could function as a complementary marker within the more extensive ACPA repertoire, possibly improving overall risk stratification when evaluated alongside established specificities [51]. This is consistent with studies suggesting that anti-MCV antibodies may be associated with severe extra-articular RA phenotypes; however, evidence for improved specificity for RA-ILD versus anti-CCP is currently insufficient and should be tested in head-to-head, assay-standardized cohorts [52]. While anti-CCP antibodies remain the most extensively validated ACPA specificity linked to both articular progression and extra-articular complications such as ILD in RA [17,19], selected studies indicate that anti-MCV antibodies may reflect broader autoimmune activation with potentially stronger or more specific associations with pulmonary involvement [32,53]. The observed associations may reflect the biological plausibility of a lung–joint axis in RA-ILD, whereby the lung serves as an early site of initiation and perpetuation of rheumatoid autoimmunity through mucosal inflammation induced by environmental exposures such as smoking. This process promotes local protein citrullination and the generation of ACPAs that can subsequently target shared citrullinated epitopes in synovial tissue, potentially contributing to both articular disease and pulmonary manifestations [41,42,43,54]. Vimentin represents a structural intermediate filament protein expressed in mesenchymal cells and pulmonary tissues, where its citrullinated form may acquire heightened immunogenicity through post-translational modification that generates neoepitopes capable of eliciting targeted autoimmune responses [44,55]. Citrullinated vimentin has been identified within pulmonary tissues in RA, consistent with its detection alongside synovial sites and supporting its potential as a shared autoantigen [43,44]. Whether anti-MCV antibodies serve merely as serological biomarkers or reflect underlying pathogenic mechanisms in RA-ILD warrants careful consideration, as current evidence predominantly supports an associative biomarker role without establishing direct causality [21,50]. Nevertheless, elevated anti-MCV levels may identify a broader autoimmune phenotype marked by enhanced citrullination and increased extra-articular involvement [32,52]. The biological plausibility of immune responses targeting citrullinated vimentin contributing to both pulmonary and synovial inflammation is supported by the detection of citrullinated vimentin peptides in bronchial and synovial tissues [44,55,56]. However, available data remain insufficient to confirm a direct pathogenic contribution of anti-MCV antibodies to the development of RA-ILD, necessitating further mechanistic studies. While anti-CCP antibodies remain the most widely studied ACPA specificity linked to RA-ILD risk and severity across multiple cohorts, they lack sufficient antigen specificity to identify the precise citrullinated targets most relevant to pulmonary involvement, and anti-MCV antibodies could furnish complementary information within the more extensive ACPA response despite the still-limited body of evidence supporting such a role [17,19,32,51,53]. Observations from multiple cohorts indicate that wider and more diverse ACPA responses arising through epitope spreading and recognition of multiple citrullinated antigens are associated with pulmonary involvement in RA and may reflect more extensive systemic autoimmunity [3,16,57], positioning anti-MCV antibodies as one potentially informative element within this expanded serological landscape.

Several autoantibody subsets beyond conventional anti-CCP antibodies have been linked to pulmonary involvement in RA. These include anti-CarP antibodies, which are independently associated with RA-ILD, and expanded ACPA repertoires that may reflect wider systemic and mucosal autoimmunity. Such findings suggest that distinct serological profiles could delineate different immunological or clinical phenotypes of RA-ILD, although the relative diagnostic and prognostic value of anti-MCV antibodies compared with these biomarkers remains incompletely defined [3,52]. Within this context anti-MCV antibodies constitute one interrelated element of the expanded ACPA repertoire that may supply complementary serological information alongside anti-CCP antibodies and other subsets such as anti-CarP antibodies thereby potentially identifying a subgroup of patients marked by intensified citrullination systemic autoimmunity and extra-articular involvement although current evidence remains too limited to delineate their precise diagnostic or prognostic role in RA-ILD evaluation [3,21,53]. Addressing this gap is particularly pressing given the substantial clinical challenges of early RA-ILD identification, where a high proportion of cases remain subclinical until advanced fibrotic changes have developed [3,35]. Reliance on respiratory symptoms alone lacks sensitivity, as up to one-third of affected patients may be asymptomatic at detection, while pulmonary function tests may miss early or mild disease and HRCT, although the diagnostic reference standard, entails radiation exposure and cost and is best used within risk-stratified screening and monitoring strategies [17,58]. Consequently, the identification of accessible serological biomarkers capable of supporting earlier detection and risk stratification has emerged as a key priority to enable timely intervention and improve outcomes. This unmet need has in turn stimulated interest in autoantibody-based candidates such as anti-MCV antibodies, which may furnish complementary information on citrullinated antigen responses linked to pulmonary involvement beyond that provided by conventional anti-CCP testing [21,50]. Anti-MCV positivity or elevated titers may help identify RA patients at heightened risk of pulmonary involvement, as suggested by the higher prevalence and levels observed in those with RA-ILD compared with RA patients without ILD [21,50]. Biomarker assessment must nevertheless be integrated with clinical history, HRCT, and PFTs rather than used in isolation, given the imperfect sensitivity and specificity of any single autoantibody and the currently limited evidence for anti-MCV in RA-ILD. Current evidence remains too limited to endorse routine screening based solely on anti-MCV antibodies, underscoring the need for larger prospective studies before any change in clinical practice can be recommended.

Anti-MCV antibodies may aid in delineating distinct clinical and immunological phenotypes of RA associated with intensified citrullination and greater extra-articular involvement [32,53], potentially enabling biomarker-driven stratification that supports individualized monitoring protocols and tailored therapeutic strategies for RA-ILD patients. Future investigations should therefore prioritize larger prospective multicenter cohorts with longitudinal follow-up to evaluate the predictive and prognostic utility of anti-MCV antibodies over time, predefine clinically relevant pulmonary outcomes, and standardize anti-MCV assay platforms, calibrators, units, and positivity thresholds, because the available studies used different ELISA systems and interpretive cut-offs. Such efforts would also facilitate integration of anti-MCV measurements with HRCT findings, pulmonary function parameters, and complementary biomarkers such as anti-CarP or expanded ACPA repertoires to refine risk stratification algorithms. Parallel mechanistic and translational studies are required to determine whether these antibodies function as pathogenic mediators of pulmonary citrullination or serve principally as serological surrogates of mucosal and systemic autoimmunity, thereby informing potential biomarker-driven approaches to individualized monitoring and therapeutic selection in RA-ILD.

### Limitations

This scoping review has several limitations. First, only two studies met the predefined eligibility criteria, limiting the breadth of the available evidence. Second, the included studies showed substantial methodological heterogeneity, particularly regarding anti-MCV assay platforms and positivity thresholds, precluding direct comparisons. Third, only English-language publications were considered, and no systematic search of grey literature was performed, which may have resulted in the omission of relevant evidence. Finally, as a scoping review, our objective was to map the available literature rather than to perform a formal quality assessment or quantitative synthesis of the included studies.

## 5. Conclusions

Current evidence suggests that anti-MCV antibodies may represent promising biomarkers in RA-ILD, as anti-MCV positivity and elevated titers appear to be associated with pulmonary involvement in RA, although the currently available evidence remains preliminary. Only a small number of studies have specifically investigated anti-MCV antibodies in RA-ILD. Despite this limited evidence base, the available findings suggest an association between anti-MCV antibodies and pulmonary involvement in RA. These observations justify further investigation to clarify their potential role. The association between anti-MCV antibodies and RA-ILD is biologically plausible within the framework of the lung–joint axis hypothesis. Pulmonary citrullination and shared autoimmune responses between lung and synovial tissues may reflect, and possibly contribute to, this relationship. These mechanisms support continued interest in anti-MCV antibodies as candidate biomarkers in RA-ILD. However, the limited number of available studies and their predominantly cross-sectional designs underscore the need for larger prospective multicenter cohorts with extended longitudinal follow-up to validate anti-MCV antibodies. Such research is required to clarify their diagnostic accuracy, prognostic value, and any potential pathogenic role in RA-ILD. Until these data emerge, anti-MCV antibodies should be considered investigational markers whose clinical utility remains to be established. In the future, if externally validated, anti-MCV antibody assessment could complement rather than replace HRCT and Pulmonary Function Tests (PFTs). While HRCT remains the reference standard for detecting RA-ILD because of its high sensitivity, anti-MCV antibodies may contribute to identifying patients at increased risk who could benefit from targeted imaging and closer longitudinal monitoring. Such a multimodal approach integrating serological biomarkers with imaging and functional assessment may improve risk stratification while avoiding unnecessary HRCT examinations in unselected patients. Additional research should also focus on elucidating whether anti-MCV antibodies are merely indicators of heightened immune activity, markers of citrullinated vimentin responses, or active participants in disease progression.

## Figures and Tables

**Table 1:** Characteristics and principal findings of the included studies that evaluated anti-MCV antibodies in RA-ILD [1]. Anti-MCV assay platforms and thresholds differed between studies. Tian et al. [50] used an ORGENTEC ELISA with a ≥ 20 U/mL positivity threshold, whereas Elsayed et al. [21] used a Cusabio ELISA with >20 U/mL positivity and reported a ROC-derived 155.5 U/mL threshold for ILD discrimination. These cut-offs should not be considered interchangeable without assay-specific calibration and external validation.

Study	Design	Population	Assay Platform/ Manufacturer	Positivity Threshold	ROC-Derived Cut-Off	Main Findings	Key Results
Tian et al., 2016 [50]	Cross-sectional study	37 RA-ILD vs. 38 RA without ILD	ELISA (ORGENTEC Diagnostika GmbH, Mainz, Germany)	≥20 U/mL	Not reported	Anti-MCV positivity and titers were significantly higher in RA-ILD, whereas anti-CCP levels did not significantly differ between groups	Anti-MCV positivity: 100% vs. 71.1%; anti-MCV independently associated with RA-ILD
Elsayed et al., 2024 [21]	Case–control study	40 RA-ILD vs. 40 RA without ILD + 40 healthy controls	ELISA (CUSABIO Biotech Co., Ltd., Wuhan, China)	>20 U/mL	155.5 U/mL (80% sensitivity, 75% specificity for RA-ILD)	Anti-MCV levels were significantly higher in RA than in healthy controls and were further increased in RA-ILD, where they correlated with disease activity and pulmonary impairment.	AUC 0.815; sensitivity 80%; specificity 75%; negative correlation with DLCO and FVC

## Data Availability

No new data were created or analyzed in this study. Data sharing is not applicable to this article.
